# Automated Classification of Seizure Onset Pattern using Intracranial Electroencephalogram Signal of Non-Human Primates

**DOI:** 10.1088/1361-6579/add9e3

**Published:** 2025-06-10

**Authors:** Fahimeh Mohagheghian, Sujin Jiang, Mark J Connolly, Ellen D Sproule, Robert E Gross, Xiao Hu, Annaelle Devergnas

**Affiliations:** 1 Nell Hodgson Woodruff School of Nursing, Emory University, Atlanta, GA, USA; 2 School of Chemical, Material, and Biomedical Engineering, University of Georgia, Athens, GA, USA; 3 Emory National Primate Research Center, Emory University, Atlanta, GA, USA; 4 Department of Neurology, Emory University School of Medicine, Atlanta, GA, USA; 5 Wallace H. Coulter Department of Biomedical Engineering, Emory University and Georgia Institute of Technology, Atlanta, GA, USA; 6 Department of Neurosurgery, Robert Wood Johnson Medical School, Rutgers University, New Brunswick, NJ, USA

**Keywords:** Intracranial EEG, Seizure, Onset pattern, Automatic Classification, Primates

## Abstract

**Objective:**

To develop and validate a machine learning framework for the classification of distinct seizure onset patterns using intracranial EEG (iEEG) recordings in a non-human primate (NHP) model of penicillin-induced seizures.

**Approach:**

iEEG data were collected from six NHPs, comprising 1,496 frontal and 549 temporal lobe seizures. Seizure onset patterns were manually categorized into five types: Sharp Activity (5–15 Hz), Low Amplitude Fast Activity (15–30 Hz), Delta Brush (1–3 Hz with bursts), High Amplitude Spike (2–5 Hz), and Polyspike. A Random Forest classifier was trained using features extracted from optimized seizure onset segments. Feature selection and seizure segment length optimization were performed using nested cross-validation to enhance classification accuracy and generalizability.

**Main results:**

The classifier achieved strong performance with F1-scores exceeding 79% for Sharp Activity, Low Amplitude Fast Activity, and High Amplitude Spike patterns. When validated on an independent temporal lobe seizure dataset, the model demonstrated robust generalizability, achieving precision and sensitivity above 80% for Sharp Activity and High Amplitude Spike.

**Significance:**

These findings demonstrate that the suggested spectral and dynamic features can effectively distinguish seizure onset patterns and generalize in distinct brain regions. Although there are limitations due to use of manual annotations and the sample size of certain categories, the proposed approach provides a framework for automatic classification of seizure onset patterns. Further, the framework has a potential use for epilepsy research and clinical applications in future.

## 1. Introduction

Epileptic seizures are characterized by abnormal or hyper-synchronous neuronal activity that originate from the neocortex or mesial temporal lobe structure [1]. Focal epilepsy represents almost 80% of epilepsy and patients are often resistant to medication [2]. The interpretation of intracranial EEG (iEEG) is key in diagnosing and treating epilepsy, especially when considering epilepsy surgery. It helps in identifying the seizure onset zone and deciding on the best surgical option [3]. Various studies have shown considerable interest in investigating the seizure onset zone and onset patterns [4–7]. The classification of seizure onset patterns is critical for understanding seizure dynamics and improving diagnostic and therapeutic approaches. Studies have revealed that a patient with a single epileptogenic focus can generate diverse seizure onset patterns and that characterization of patterns through iEEG could serve as an additional feature to clinically guide effective seizure management [8, 9]. Specific seizure onset patterns have correlated with the type and severity of underlying neuronal abnormalities [10, 11], while others have been associated with the intrinsic anatomy or network connectivity of a specific brain region [5, 12]. How different seizure onset patterns are associated with varying network interactions and propagation pathways is a topic of considerable scientific interest and can significantly advance our understanding of the mechanisms underlying seizure generation and propagation [13–16]. However, such studies require a large number of seizures which are difficult to obtain from patients. The model of penicillin (PCN) induced seizure in non-human primates (NHP) creates a series of on demand seizures that reliably mimic human seizure dynamics [17–19]. Previous studies have investigated the automatic classification of seizure types and onset patterns using patients intracranial or scalp EEG recordings. For instance, Wu et al. introduced a classification framework that combined multi-class specific bands common spatial pattern with joint approximate diagonalization for enhanced spatial filtering [20]. In their study, features extracted from intrinsic mode functions (IMFs) were used to capture nonlinear and non-stationary EEG dynamics and feature selection was performed using linear discriminant analysis and random forest. This approach, achieved an accuracy of 96.14% and an F1-score of 0.9679, demonstrating strong potential for multiclass seizure classification. Another study using a support vector machine (SVM) classifier achieved an overall classification accuracy of 80.7%. This high performance shows the capability of the proposed model for detecting various seizure onset patterns similarly to visual classification [21]. In another study, EEG signals were transformed into spectrogram stacks and processed via convolutional neural network (CNN), using both direct transfer learning and feature extraction followed by SVM classification. Results of this study demonstrated the superiority of CNN-based approaches over conventional feature-driven methods for seizure type classification [22].

To the best of our knowledge, no prior studies have addressed the classification of seizure onset patterns in animal models. These models are important for understanding of the mechanisms underlying the seizure initiation and propagation. The primary objective of this study is to develop and validate a machine learning-based framework for the robust classification of seizure onset patterns using iEEG recordings. We leverage computational methods to reliably characterize different types of seizure onset patterns observed in the NHP model of PCN-induced seizure in the frontal and temporal lobe. Seizures are initially manually categorized following a patient seizure pattern classification [23]. The performance of the machine learning model is then validated to ensure robustness and reliability of the model. Using this comprehensive animal dataset, we propose a framework for developing a model with a potential for future validation on human dataset. This framework can be utilized in clinical settings to improve and individualize the patients’ seizure detection and classification.

## 2. Methods

### 2.1. Data Collection

#### 2.1.1. General Experimental Strategy

We recorded iEEG activity during frontal lobe seizures and temporal lobe seizures. Seizures were induced by local injection of PCN in either the motor cortex or the hippocampus (HPC) and recordings were performed at the seizure focus. Seizures were manually detected and categorized based on their onset patterns. An automatic classifier was trained using a training set and tested on an independent set of features extracted from seizures onset.

#### 2.1.2. Animals, Surgical Procedures

Six rhesus monkeys (macaca mulatta; 6-10 kg; three males and three female) were used in this study. We induced frontal lobe seizures on NHP 1-3 and temporal lobe seizures on NHP 4-6. The animals were pair-housed with *ad libitum* access to food and water. We confirm that we have read the Journal’s position on issues involved in ethical publication and affirm that this report is consistent with those guidelines. This study was performed under the Emory IACUC approval PROTO201700590, PROTO201700565, PROTO202200115, and PROTO202200091. All experiments were performed in accordance with the United States Public Health Service Policy on the humane care and use of laboratory animals, including the provisions of the “Guide for the Care and Use of Laboratory Animals” [24]. All studies were approved by the Institutional Biosafety and Animal Care and Use Committees of Emory University.

The animals were first conditioned to be handled by an experimenter and to sit in a primate chair. They then underwent aseptic surgery under isoflurane anesthesia (1-3%) for implantation of a recording chamber (Crist Instruments, Hagerstown, MD; inner chamber diameter 16mm). The chamber was placed to either access the frontal cortex (NHP 1-3) or the HPC (NHP 4-6). The animals with the HPC access also had a microdeep depth stereo-EEG electrode-5 contact (DIXI medical) chronically implanted in the anterior part of the HPC.

#### 2.1.3. Seizure Induction

Seizures were induced by intracerebral injection of PCN diluted into sterile water (PCN G sodium salt, and sterile water from Sigma-Aldrich, St. Louis, MI). For seizure induction, we used a final concentration of either 1,000 or 2,500 unit of PCN /μl and an average dose of 0.5-3μl was injected in either the arm region of the primary motor cortex or the HPC. The drug was delivered at a rate of 1 μl/min, using an injection system connected to a mechanical pump and a Hamilton syringe (CMA, Harvard Biosciences Inc, Krista, Sweden). The injection system was lowered into the area of interest (primary motor cortex or HPC) and after 10 min the injection started. Five minutes after injection, the system was clamped to avoid PCN leakage and carefully raised. Details of the model have been previously described [19, 25, 26]. In this model of on-demand seizure, PCN injection induced several focal seizures over a period of 4-6 hours. No generalization was observed, and no spontaneous seizures were detected between injections. Frontal lobe seizures were characterized by tonic-clonic movement of the arm and temporal lobe seizures were not associated with obvious clinical motor symptoms (no systematic swallowing or chewing were noted during these temporal lobe seizures). We induced a total of 38 frontal lobe seizures sessions and collected a total of 1,696 seizures. For the temporal lobe seizures, we induced 40 seizure sessions and collected 673 seizures (Table1). Seizure induction sessions were at least one week apart.

#### 2.1.4. Frontal and Hippocampal iEEG

For all NHPs, recordings were made while the animal sat in a primate chair with its head immobilized but with body and limbs free to move. Before each frontal lobe seizures session, a concentric bipolar electrode (FHC, US; 0.5/0.5/0.5) was lowered next to the injection site and bipolar signal was recorded using a PZ5 system (Tucker-Davis Technologies, US). For temporal lobe seizures, recordings were performed using the stereo-EEG implanted in the HPC during the surgery connected to a Cerebus system (BlackRock Microsystem, US). For both systems, iEEG signals were sampled at 2 kHz and stored with the corresponding synchronized video of the animal.

### 2.2. Seizure Onset Pattern Categorization

**Figure 1.**
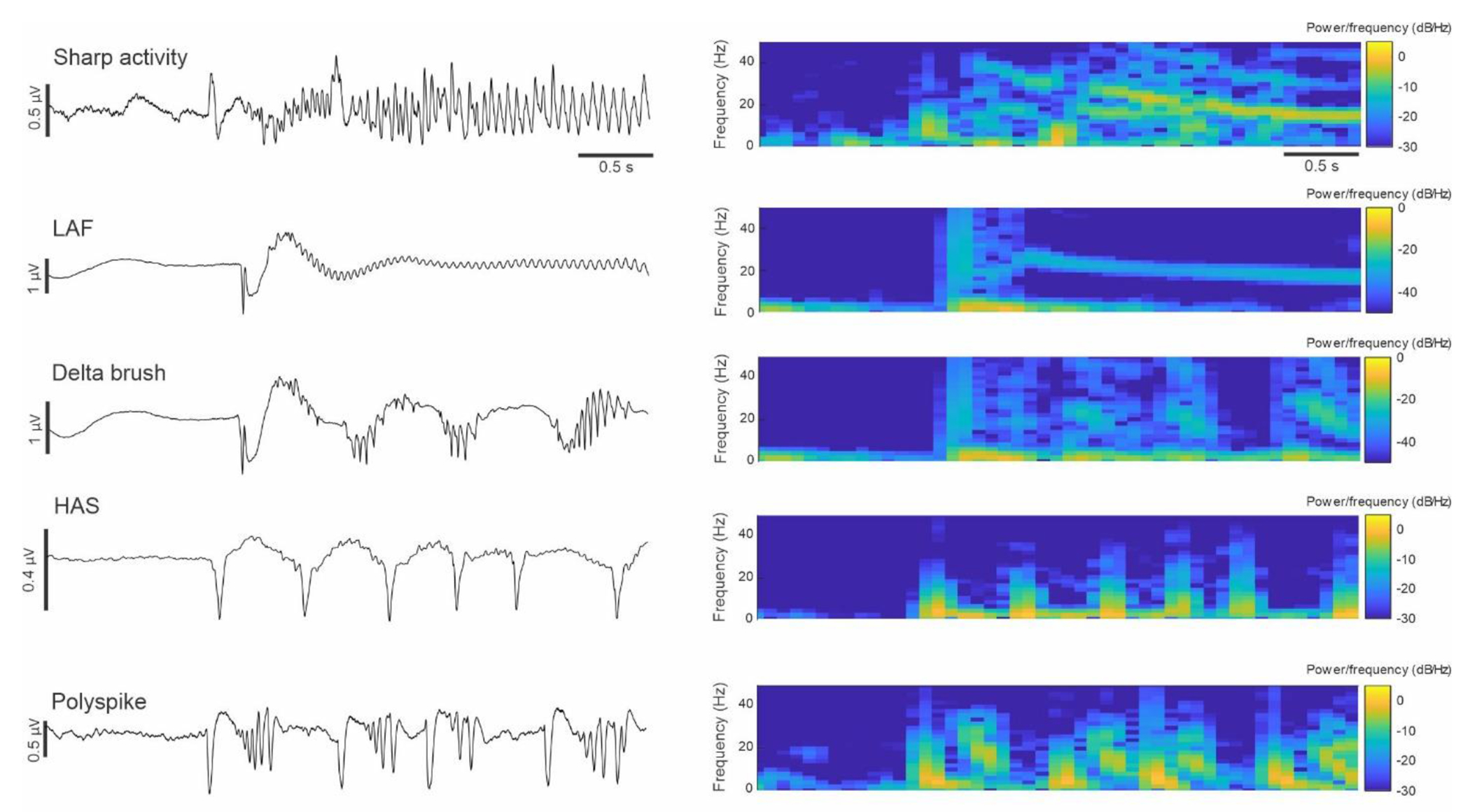
Examples of typical seizure onset patterns. Sharp Activity was characterized by sharp-contoured, medium-voltage activity with mixed frequencies. The LAF pattern featured low-amplitude rhythmic activity between 15 and 30 Hz. The Delta brush pattern consisted of waves at 1–2 Hz with superimposed bursts at 10–30 Hz. The HAS pattern was defined by high-voltage spike complexes around 3 Hz, with a spike marking the seizure onset. The polyspike onset pattern consisted of a high-amplitude spike followed by polyspike bursts lasting ~0.5 seconds, accompanied by voltage attenuation.

Data were imported into the Spike 2 interface (CED, Cambridge, UK) for offline annotation of ictal activity. Seizure onset and offset were manually marked, and patterns were categorized by two trained experimenters based on the morphology and spectral characteristics of the first three seconds of recorded activity in the ictogenic zone. In cases of disagreement, a third more senior experimenter provided the final decision, ensuring consistency and minimizing potential bias in classification. The inter-annotator agreement, calculated using Cohen’s kappa (κ), was 0.51. A total of 336 seizures required intervention from a third annotator to resolve disagreements. Based on the onset patterns previously described in human studies [15, 23, 27], we identified five distinct seizure onset patterns in our NHP model (Figure 1):

- Sharp Activity: Medium-frequency oscillatory activity characterized by low-to-medium voltage sharp spikes in the 5–15 Hz range.
- Low Amplitude Fast Activity (LAF): Low-voltage rhythmic activity in the 15–30 Hz range.
- Delta Brush: Low-frequency waves between 1 and 3 Hz, accompanied by bursts of higher activity.
- High Amplitude Spike (HAS): Spikes or spike complexes occurring at seizure onset with a frequency between 2 and 5 Hz.
- Polyspike: High-amplitude polyspike bursts lasting 0.5–1 s, followed by low-amplitude suppression lasting 0.2–0.5 sec, with a primary frequency of approximately 20 Hz.

Of the 2,350 recorded seizures, 2,045 high-quality seizures were categorized (Table 1). Seizures with poor signal-to-noise ratios or artifacts were excluded from analysis. After manual classification, the polyspike pattern was removed from automatic classification due to an insufficient number of samples.

### 2.3. Machine Learning-based Classification

**Figure 2.**
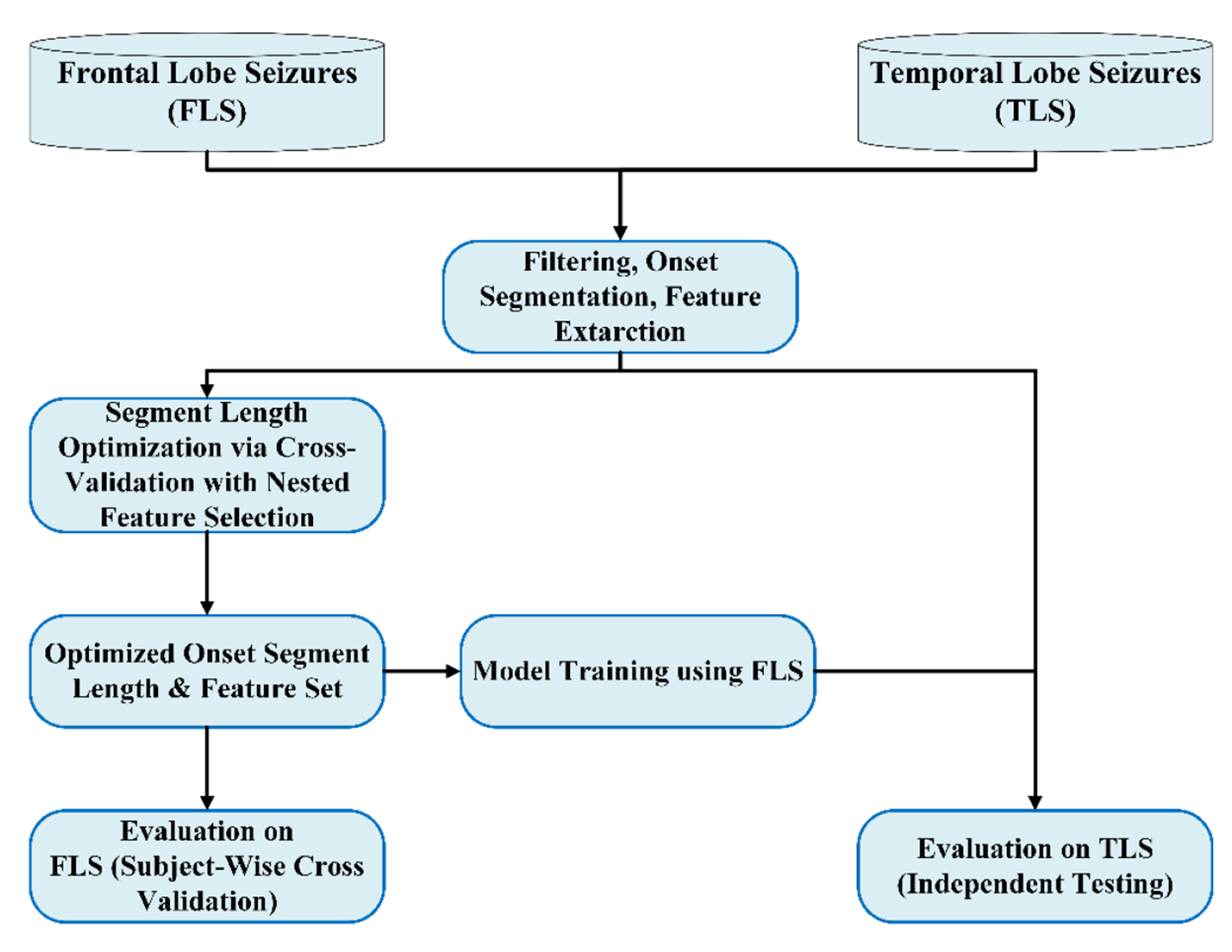
Overview of the machine learning-based seizure onset classification workflow. Raw iEEG signals were recorded during temporal and frontal lobe seizures. Data preprocessing and feature selection were followed by seizure onset segment length optimization. Nested cross-validation was applied to determine the optimal feature set. The classifier was trained on frontal lobe seizures using the optimized segment length and features, with its generalization assessed on independent temporal lobe seizure data and validated through subject-wise cross-validation on frontal lobe seizures.

Workflow for the machine learning-based seizure onset classification methodology is represented in Figure 2. Raw iEEG signals were recorded during frontal and temporal lobe seizures. For the frontal lobe seizures dataset, data preprocessing was followed by optimization of the seizure onset segment length and the application of a nested cross-validation approach to select the optimal feature set.

In the Model Evaluation stage, the classifier was trained using the frontal lobe seizures dataset, and its performance was assessed in two distinct scenarios: (1) using the temporal lobe seizures dataset as an independent test set to evaluate the model’s generalizability across seizures originating from a different brain region, and (2) using subject-wise cross-validation on the frontal lobe seizures dataset to assess intra-dataset performance. This workflow highlights the robustness of the proposed approach in adapting to diverse seizure types and anatomical regions.

#### 2.3.1. Intracranial EEG Preprocessing and Seizure Onset Segmentation

All subsequent data analyses were performed using MATLAB R2023a. The seizure signals were filtered using a finite impulse response (FIR) filter with a 60 Hz cutoff frequency and down-sampled to 120 Hz. To effectively remove power line noise artifacts at 60 Hz, an infinite impulse response (IIR) comb notch filter with the 0.3 Hz bandwidth of the filter notch at −3 dB was applied.

Each seizure onset was then divided into five different segments: 1) the 2 seconds preceding the seizure onset and the first 2 seconds of the seizure, 2) the first 5 seconds of the seizure, 3) the first 4 seconds of the seizure, 4) the first 3 seconds of the seizure, and 5) the first 2 seconds of the seizure. Subsequent processing steps were applied to each segment, and the performance of the classifier model was evaluated across these segmentations to identify the optimal seizure onset segment length for further analysis.

#### 2.3.2. Feature Extraction

Feature extraction was performed to derive the relevant characteristics of the iEEG signals, including time-domain, frequency-domain, and time-frequency information. These features were selected to enhance the performance of the classification model by capturing the dynamic characteristics of the signal.

For time-domain analysis, features were extracted to capture both statistical properties and signal complexity. The extracted features included variance, skewness, kurtosis, coefficient of variation, interquartile range, and the 5th and 95th percentiles of the time-domain signal. Skewness quantifies the asymmetry of the distribution of signal’s amplitude. It shows whether the distribution is biased toward the positive or negative values. Kurtosis, a measure of the distribution’s ‘tailedness’, indicates how frequently the extreme values occur. The coefficient of variation, which is calculated as the ratio of the standard deviation to the mean, is the normalized variability of the signals with different scales. Interquartile range measures the spread of the middle 50% of the signal values. The 5th and 95th percentiles define the amplitude thresholds that encompass 90% of the signal. All these computations were performed using built-in MATLAB functions to ensure consistency and accuracy.

The extracted complexity measures included Higuchi fractal dimension and Shannon entropy. Higuchi fractal dimension calculates the complexity of brain activity by measuring the degree of self-similarity in the iEEG signal. Higher Higuchi fractal dimension values show more complex and irregular signal, while lower values indicate more predictable and regular patterns in the signal. Shannon entropy measures the randomness or complexity of the signal by quantifying the uncertainty in the signal fluctuations during the time. Higher entropy values reflect greater irregularity and complexity in the iEEG signal.

The discrete wavelet transform (DWT) provides an efficient time-frequency representation with low computational complexity. In this study, we applied a Daubechies-4 wavelet in four decomposition levels, yielding four frequency bands: [3.75–7.5 Hz], [7.5–15 Hz], [15–30 Hz], and [30–60 Hz]. From the wavelet decomposition, the coefficient of variation of the detail coefficients in each frequency band and the total wavelet energy across all detail coefficients were extracted to characterize seizure onset patterns.

For frequency domain features, power spectral density (PSD) was estimated using the Welch method with a Hamming window (50% overlap) and a fast Fourier transform (FFT) length of 256, chosen as the maximum of 256 and the next power of 2 of the window length. Thus, the spectral resolution of 0.47 Hz, calculated as:

(1) $$\mathrm{Frequency resolution}=\frac{f_{s}}{N_{fft}}$$

where *f_s_* and *N_fft_* are the sampling frequency and FFT length, respectively.

Features extracted from the PSD included mean and median frequencies, along with the relative power of four frequency sub-bands: Delta ([0.5–4] Hz), Theta ([4–8] Hz), Alpha ([8–12] Hz), and Beta ([12–30] Hz). Further, statistical measures from spectral entropy, spectral skewness, spectral kurtosis, and spectral flux were computed to characterize the dynamic properties of the frequency spectrum. The mathematical formulations for these measures are presented below (Equations 2–6):

(2) Spectral entropy=−∑f=0fs2P(f)log2[P(f)]

(3) Spectral skewness=∑f=0fs2P(f)(f−μf)3(∑f=0fs2P(f)(f−μf)2)3/2

(4) Spectral kurtosis=∑f=0fs2P(f)(f−μf)4(∑f=0fs2P(f)(f−μf)2)2

where *P*(*f*) is the normalized PSD at frequency *f,* and *μ_f_* is the mean frequency, given by:

(5) μf=∑f=0fs2fP(f)

(6) Spectral flux=∑f=0fs2(Pt(f)−Pt−1(f))2

where *P_t_*(*f*) is the normalized PSD at frequency *f* and time frame *t*.

Spectral entropy quantified the complexity of the signal, while spectral skewness measured the asymmetry of the spectrum compared to its centroid. Spectral kurtosis provided insights into transients within the signal. Spectral flux captured dynamic frequency variations by measuring the rate of change in the power spectrum across consecutive time frames. In total, 48 features were extracted and used for subsequent analysis.

#### 2.3.3. Random Forest (RF) Algorithm Overview

The RF method was employed to identify the most significant features for seizure onset classification. RF is a supervised learning algorithm that integrates decision trees with the bagging (Bootstrap Aggregation) method. RF was chosen due to its robustness to handle imbalanced datasets and small sample sizes in each class. The core concept of RF involves resampling the training dataset through a procedure called “bootstrap”. In bootstrap process, each decision tree is trained on a randomly selected subset of the feature set and samples. The optimization of RF hyperparameters is performed automatically during model training to maximize classification performance. Finally, predictions from individual trees are aggregated to produce the final classification results.

#### 2.3.4. Optimization of Segment Length and Feature Selection

A systematic approach was used to optimize both the seizure onset segment length and feature selection. Subject-wise 3-fold cross-validation strategy was applied to different onset segment lengths using the frontal seizure dataset. In each fold, seizures from one monkey were employed as the test set, while data from the two remaining subjects were used to train the model.

To prevent data leakage, feature selection was performed within a nested cross-validation framework, ensuring an optimal feature set specific to each segment length. A RF-based method was used to determine the feature importance. Features were retained based on their frequency of selection across cross-validation iterations, yielding the final feature set for each segment length and ensured robustness and generalizability for all subjects.

The optimal segment length and corresponding feature set were identified by evaluating the area under the curve (AUC) of receiver operating characteristic (ROC) curves. This approach minimized the risk of overfitting and ensured a high-performance final model.

#### 2.3.5. Model Training, Evaluation, and Generalization

Following segment length optimization and feature selection, an RF classifier was trained to classify seizure onset into four distinct classes. A subject-wise 3-fold cross-validation strategy was applied to frontal lobe seizure data using both the optimized segment length and selected feature set to ensure robust training and validation.

To assess generalization, the model was retrained on the entire frontal lobe seizure dataset and evaluated on an independent test set consisting of temporal lobe seizures. This strategy provides the model evaluation by testing the model on previously unseen anatomical regions.

## 3. Results

### 3.1. Seizure Onset Pattern in Frontal and Temporal Lobe Seizures

**Figure 3.**
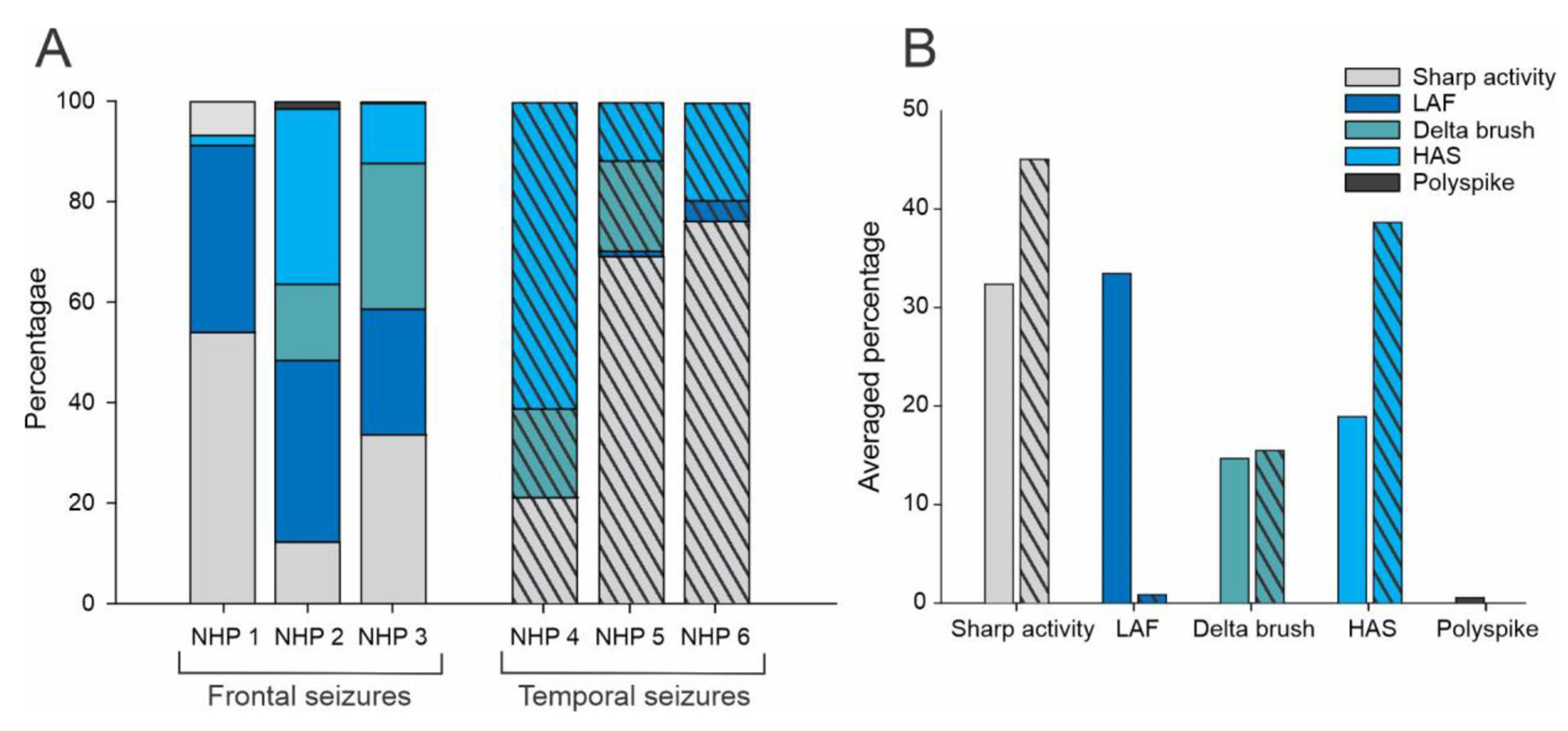
A. Distribution of seizure onset pattern for each animal, for frontal seizure (NHP 1-3) and temporal lobe seizure (NHP 4-6). B. Comparison of distribution of seizure onset pattern averaged across animals for frontal seizure (solid colors) and temporal lobe seizure (dashed colors)

We analyzed 1496 high quality frontal lobe seizures collected from the motor cortex of 3 NHPs (Table 1 and Figure 3A). Overall, LAF and Sharp Activity onset pattern were equally represented with a prevalence of respectively 33.42%, 32.35%. HAS and Delta brush patterns were observed in 18.92% and 14.70%, and Polyspike in only 0.6% of the sample. Notably, we found significant differences in the distribution of seizure pattern between the 3 NHPs (Chi-Square Test of Independence=80.27, p<0.001). Thus, for NHP2 the proportion of Sharp Activity only reached 12.19% while reaching 53.97% and 33.57% respectively for NHP 1 and NHP 3.

For temporal lobe seizures, we also included 3 NHPs, however the seizures were less frequent, and we could only analyze 549 high quality seizures (Table 1 and Figure 3A).

We observed a similar prevalence of Sharp Activity and HAS onset patterns with a rate of 44.99% and 38.61% respectively, averaged across the 3 NHPs. For temporal lobe seizures the Delta brush pattern was more represented than LAF with 15.48% and 0.91% respectively. No Polyspike pattern was found in our temporal lobe seizure sample. Similarly to frontal lobe seizure we found significant difference between the 3 animals exhibiting temporal lobe seizure (Chi-Square Test of Independence=101.1, p<0.001).

We also found a difference in the proportion of onset pattern between frontal lobe seizures and temporal lobe seizures (Chi-Square Test of Independence=40.2, p<0.001). For frontal lobe seizures, the 2 dominant patterns were Sharp Activity and LAF while for temporal lobe seizures, Sharp Activity and HAS onset patterns were the dominant one (Figure 3B).

### 3.2. Classification Performance

#### 3.2.1. Optimization of the Onset Segment and Feature Set

To determine the optimal seizure onset segment length and feature set, we trained the model using various segment durations. Given the limited number of temporal lobe seizures for each class, this analysis was conducted exclusively on frontal lobe seizures. A leave-one-subject-out cross-validation approach was applied to each of the five segment lengths (see Section 2.3.1) to systematically evaluate and compare classifier performance.

**Figure 4:**
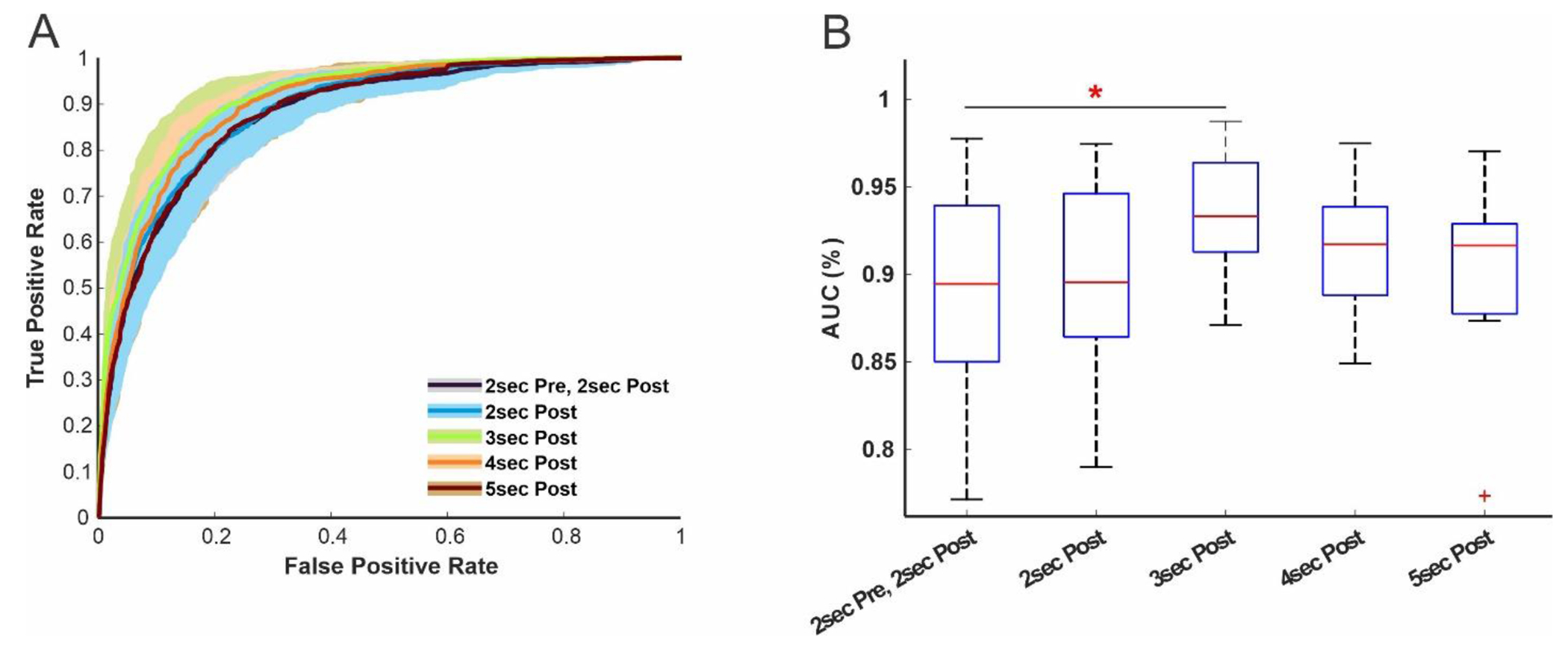
A. Averaged ROC curve across all classes obtained from various segment lengths. The shaded areas represent the standard deviations around the mean ROC curves. B. Boxplots of AUC values across different seizure onset segment lengths for all onset pattern classes. A significant difference (Friedman test, P < 0.05, indicated by *) was observed between the “2sec Pre, 2sec Post” and “3sec Post” conditions, highlighting the impact of segment length on classification performance.

**Figure 5.**
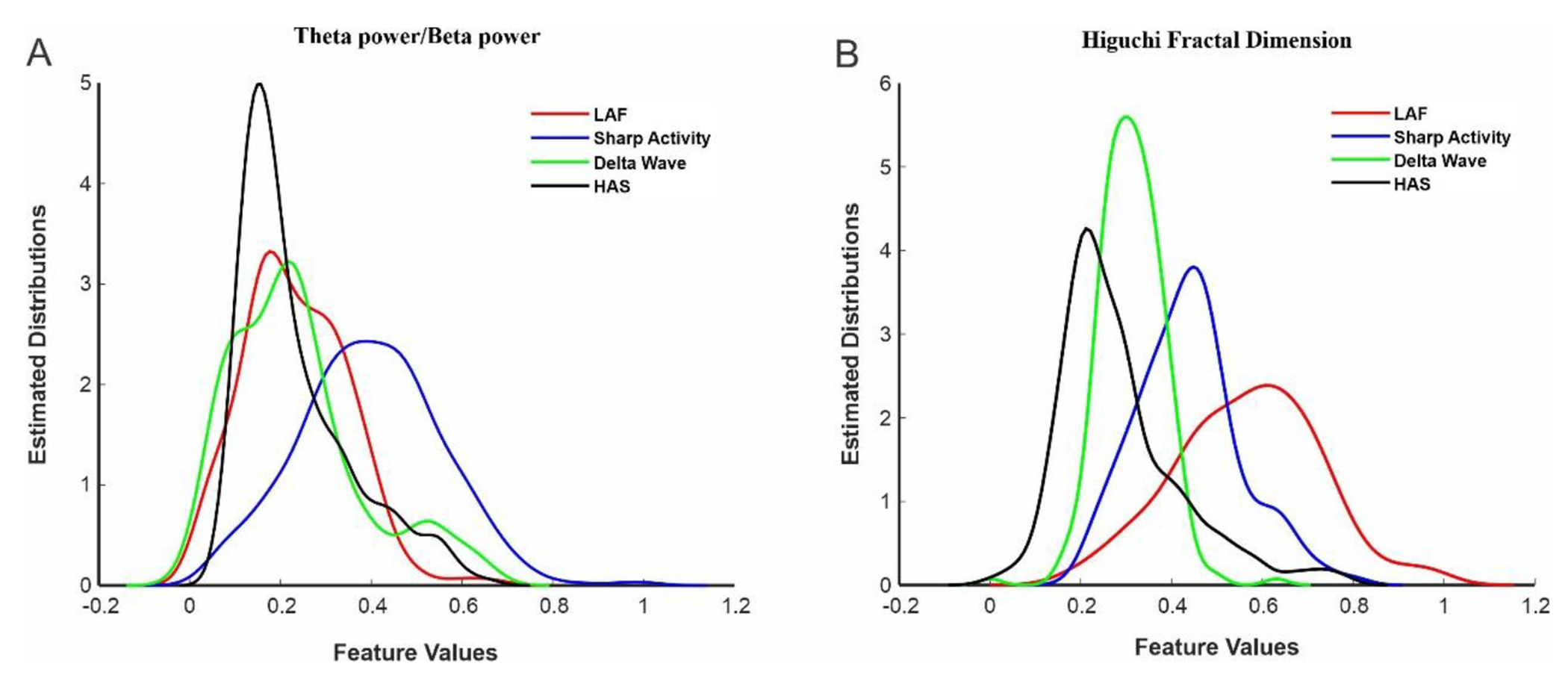
Estimated probability density distributions of feature values for two selected features: relative power of Theta to Beta band (A), and Higuchi fractal dimension, across all seizure onset pattern classes (B). Different colored curves represent the distributions for each class, illustrating the variation in feature values among the classes.

**Figure 6.**
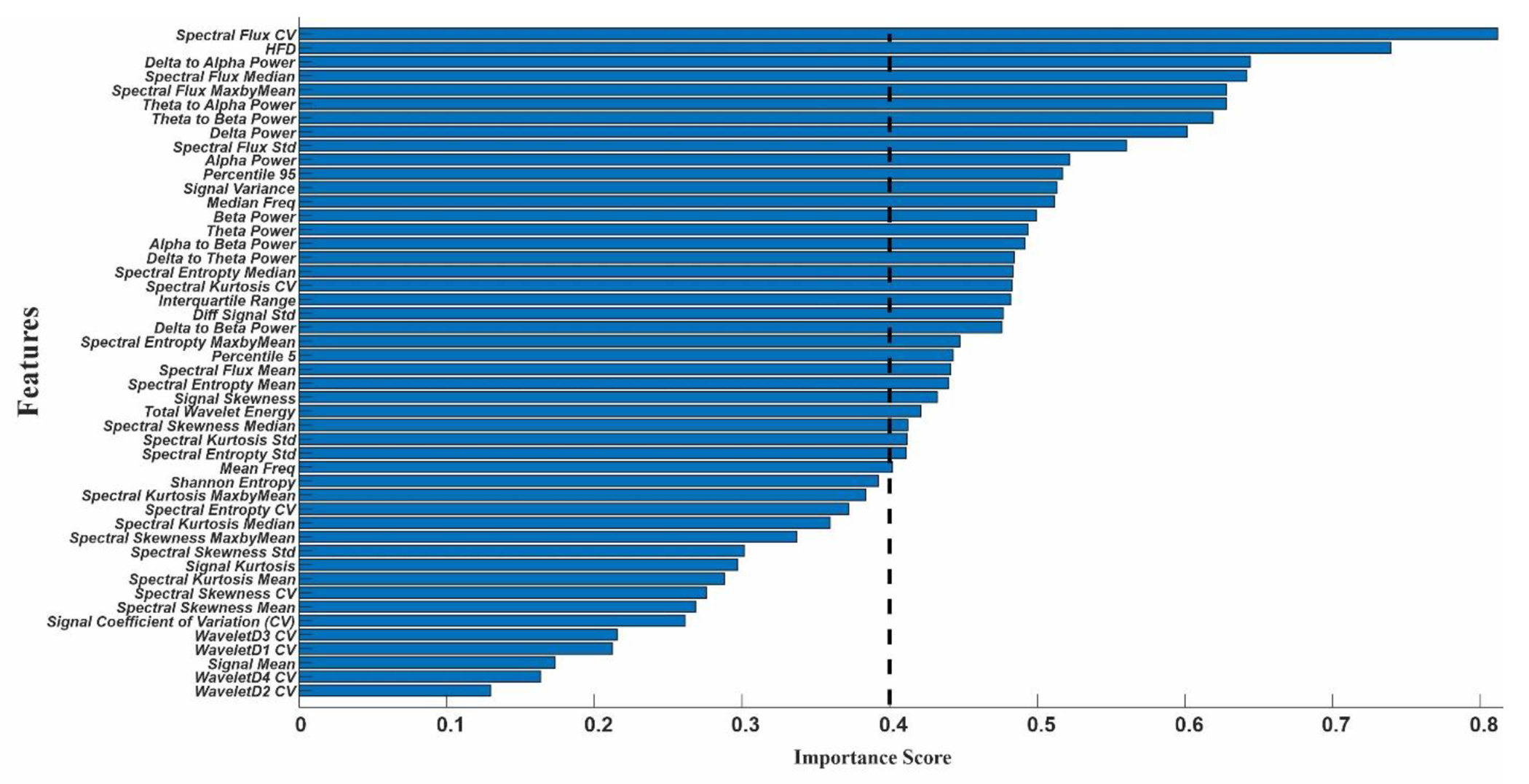
Feature importance scores and the threshold derived from the RF feature selection approach across 3-fold subject-wise cross-validation (CV: Coefficient of Variation).

Table 2 summarizes the per-class AUC values of ROC curves for various segment lengths. Figure 4A presents ROC curves averaged across classes for different segment lengths. The shaded areas represent the standard deviations around the mean ROC curves. The model trained on the first 3 seconds of seizure onset achieved the highest classification performance across all seizure onset classes. Furthermore, AUC values across various onset segment lengths are illustrated using boxplots in Figure 4B. A statistically significant improvement (Friedman test, P < 0.05) in AUC distributions was observed between the 3-second post-onset segment and 2-second pre-post combination. Consequently, subsequent analyses were conducted using the initial 3 seconds of seizure data, along with the corresponding optimized feature set. Figure 6 (Appendix I) illustrates the averaged feature importance scores across all folds and the selection threshold. The threshold of 0.4 was identified based on the features that were selected at least once across the cross-validation folds using the RF feature selection approach. Based on this criterion, 32 out of the 48 total features were retained, which reflect their consistent contribution to classification performance. The selected features included interquartile range, Higuchi fractal dimension, relative power ratios (Delta to Theta, Alpha, and Beta), mean frequency, and spectral skewness and kurtosis. Figure 5 illustrates the estimated distributions of feature values for two selected features—the relative power of Theta to Beta and Higuchi fractal dimension—across all seizure onset pattern classes, highlighting their variability and discriminative potential for classification.

#### 3.2.2. Classifier’s Performance

The performance metrics of the classifier for frontal lobe seizures are summarized in Table 3. Model evaluation included accuracy, sensitivity, specificity, positive predictive value (PPV), and F1-score. Additionally, True positives, True negatives, False positives, and False negatives are reported to provide details of classification performance.

The results demonstrate variability in performance across the four classes. HAS achieved the strongest performance, with a PPV of 87.41%, sensitivity of 88.34%, and specificity of 97.01% reflecting its reliable classification. LAF and Sharp Activity classes also performed well, with PPVs of 78.13% and 81.86%, sensitivities of 85.00% and 76.45%, and specificities of 87.94% and 91.82% respectively, indicating robust but relatively lower performance compared to HAS.

The Delta brush class exhibited the lowest classification performance, with a PPV of 72.20%, sensitivity of 67.27%, and specificity of 95.50% indicating challenges in detecting this specific onset type. Despite these variations, the overall accuracy across all four classes was 80.23%. These results demonstrate the classifier’s effectiveness for all seizure types. However, there is potential for improvement, particularly for the Delta brush class.

#### 3.2.3. Performance on Independent Test Dataset

To assess the generalizability and robustness of the classifier, we evaluated its performance on an independent test dataset consisting of temporal lobe seizures. These seizures were distinct from the training set, which was comprised of frontal lobe seizures. The independent test set allowed us to evaluate the model’s ability to generalize across different seizure types and recording locations.

As detailed in Table 4, the classifier’s performance varied significantly across the four seizure types. For Sharp Activity seizures, the model achieved the highest PPV (92.99%) and F1-score (86.33%), demonstrating strong detection accuracy with minimum false positives. In contrast, the LAF class showed perfect PPV and specificity (100%), meaning it entirely avoided false positives, but its sensitivity was low (40%), resulting in a moderate F1-score (57.14%). For Delta brush seizures, the model struggled to achieve consistent detection. Although it showed high specificity of 87.93%, lower performance in temporal lobe reflected in a low F1-score (53.13%) caused by reduced sensitivity (60%) and relatively high false-positive rates. The HAS class exhibited a more balanced performance, with a sensitivity of 86.32% and specificity of 87.24%, resulting in a high F-measure of 83.56%.

Overall, the model achieved an accuracy of 79% across all classes. The imbalanced representation of seizure types in the test set may explain the lower performance observed for the LAF and Delta brush groups, as these groups may lack sufficient positive sampled to support robust classification.

## 4. Discussion

Our study aimed to evaluate the feasibility of applying advanced machine learning techniques for automated classification of seizure onset patterns from iEEG recordings. We utilized computational methods to automatically characterize distinct onset patterns in a NHP model of penicillin-induced seizures, including frontal and temporal lobe seizures. This approach can support development of personalized treatments and improve patient care.

### 4.1. Relevance of the NHP Model for Onset Pattern Study

Patients experiencing temporal lobe epilepsy predominantly feature LAF and HAS seizure onset patterns [4, 28–30]. Frontal lobe seizures, which are less studied, have also been reported to present LAF and rhythmic slow spike activity (equivalent to our HAS) as dominant onset patterns [9]. While the Sharp Activity was the most represented onset pattern in our temporal and frontal seizure NHP model, we were still able to record a significant number of seizures with LAF and HAS onset patterns.

While similar seizure onset patterns have been observed in rodent models, e.g., LAF and hypersynchronous-onset pattern (equivalent to our HAS) in the rat pilocarpine model [19], the NHP model provides unique advantages. Notably, the variety of seizure onset patterns identified in our NHP model indicates its value for studying the dynamics and network propagation of different seizure patterns. Unlike rodent models, NHPs have larger and more complex brain structures. Their neuroanatomy and connectivity closely resemble the humans. This similarity enhances their use for examining network propagation and region-based dynamics that are more representative of human epilepsy [31–33].

Moreover, genetic and neurophysiological similarities of NHPs to humans makes them appropriate for studies on epileptogenesis and the neurobiological mechanisms underlying diverse seizure onset patterns. NHP data can consider a valuable translational model to the human data due to the similarities in cortical organization, seizure dynamics, and electrophysiological biomarkers between primates and humans [34–36]. This model provides a diverse human seizure onset patterns to investigate the neurophysiological and propagation mechanisms of seizures [15].

### 4.2. Optimized Segment Length and Feature Selection

During seizure onset, the brain undergoes rapid and dynamic changes in electrical activity. Thus, it is critical to determine the optimal data segment that captures these changes in an automatic classification. Our manual class adjudication focused on the first 3 seconds of seizure activity. This approach is consistent with prior studies which have used windows of 3-10 seconds for seizure onset classification [37]. To identify the optimal segment length, we evaluated classifier performance using different segment length, including the segments incorporating pre-ictal information. However, adding pre-ictal data did not improve classification performance. Our results demonstrated that the first 3 seconds of ictal activity contains the most relevant information for distinguishing seizure onset patterns. The first 3-second segment likely contains temporal dynamics from patterns, such as high-amplitude slow activity, high-frequency oscillations, or bursts. Our findings illustrated the discriminative characteristics of the early ictal phase and validated our approach for segment length optimization.

Comparing with prior studies, our study introduced a comprehensive set of features, incorporating time-domain, frequency-domain, and time-frequency features to effectively characterize the nonlinearities in iEEG signals. Time-domain features, such as skewness, kurtosis, and interquartile range, captured transient signal variations. In addition, frequency-domain features, including spectral entropy, spectral skewness, spectral kurtosis, and relative power across frequency sub-bands, captured oscillatory activity patterns. Integration of these diverse features significantly enhanced the classifier’s ability to distinguish between seizure onset patterns.

Our feature extraction focused on identifying discriminative features to support robust and accurate seizure classification. The final feature set selected in our analysis was consistent with those reported in previous studies, including time-domain and entropy-based features. Complexity measures such as multiscale entropy combining with frequency and time-domain features were previously used to enhance seizure onset pattern classification [20, 21]. Alternatively, Song et al. extracted Mahalanobis similarity-based and sample entropy-based features from discrete wavelet transform coefficients to classify interictal and ictal EEG segments [38].

However, unlike many prior studies that used predefined feature sets [21, 38], we employed nested cross-validation. This strategy ensured that feature selection was performed in the separated training and testing data to prevent data leakage. Therefore, the selected features enhanced robustness of the model and reduced risk of overfitting [39].

Nonetheless, variations in model performance for different onset patterns suggest that certain classes are more prone to overfitting. A decrease in F1-score observed for LAF and Delta Brush patterns in temporal lobe seizures indicates that these patterns are probably more sensitive to region-based variations. However, more samples are needed to confirm this observation.

Further, the computational efficiency of the proposed RF model was evaluated in terms of training time and complexity. Using 152 trees optimized during hyperparameter tuning and trained on 1,496 samples, the model required approximately 18 minutes for training on a computer with a CPU up to 4.80 GHz. Once trained, the preprocessing, feature extraction, and classification of a single seizure segment was performed in approximately 100 milliseconds. The computational complexity of the RF model is $O(M\times log(M)\times N_{\text{trees}}\times N_{\text{features}})$, where M is the number of samples, and $N_{\text{trees}}$ and $N_{\text{features}}$ are the number of trees and features, respectively. Thus, the resulting complexity of our model was approximately O(76,744,941) calculated using logarithm of base 2. The model satisfies latency requirements for real-time seizure onset type classification. Accordingly, deployment of the model is feasible for real-time monitoring applications.

### 4.3. Generalization of the Classifier from Frontal to Temporal Seizure

Evaluation of the classifier on an independent test dataset demonstrated the model’s ability to generalize across seizure types and anatomical regions. Several studies have suggested that distinct seizure onset patterns may share common underlying physiological mechanisms. This might occur even when they originate from different brain regions or pathological substrates. For instance, Perucca et al. [23] reported that most intracranial EEG seizure-onset patterns, including Delta brush and low-voltage fast activity were observed in multiple types of epileptogenic lesions. These findings support the hypothesis that biologically distinct pathologies can affect similar seizure-generating networks or mechanisms.

Despite the neuroanatomical and functional differences in seizures origin, the classifier exhibited robust performance in detecting Sharp Activity and HAS patterns. The high model performance suggests that the features identified for these two patterns in the frontal lobe seizures capture the same spectral and temporal characteristics of the temporal lobe seizures. Lower performance for other patterns may be influenced by region-based characteristics or dataset imbalances. Thus, the strong generalizability of the Sharp Activity and HAS patterns indicated the shared electrophysiological features of these seizures across different brain regions.

The proper generalization from frontal to temporal lobe seizures confirms the onset segment length optimization and feature selection approach. Temporal lobe seizures often involve complex neural networks, including the HPC and surrounding limbic structures, which differ from the cortical networks usually involved in frontal lobe seizures. Frontal lobe seizures usually exhibit more sudden motor manifestations [40, 41]. Consistent performance of the classifier in these diverse manifestations demonstrates its ability to detect the seizure dynamics, such as synchronous neuronal firing and pathological oscillatory patterns. This adaptability is critical for clinical applications, where seizure patterns can widely change based on etiology and patient demographics.

Previous studies have performed internal validation approaches which limit their ability for generalizability [21, 42, 43]. For instance, Makaram et al. employed 3-fold cross-validation strategy but did not specify origin of the recorded signals, leaving ambiguity about whether training and validation sets were truly independent [21]. Similarly, Aarabi and He used random subsampling of signals from different brain regions for training and testing, which may introduce overlap and fail to evaluate generalization across distinct anatomical regions [42]. By contrast, our classifier was validated on an unseen test set of temporal lobe seizures recorded from independent subjects to test the model generalization. This approach ensures a clear evaluation of the model across different seizure origins and subjects.

As epilepsy treatment becomes more personalized, robust models can help interventions based on each patient’s unique brain activity during seizures. However, addressing underrepresentation of certain seizure types in test dataset is a priority for future research. Using additional balanced datasets recorded from different brain regions can enhance the model’s generalization and provide more understanding of neurophysiological basis underlying different seizure types.

### 4.4. Limitations

Despite the promising results, this study has a few limitations. One important limitation is manual annotation of onset patterns, which introduces potential subjectivity and human error. Although annotation was performed with strict criteria to ensure consistency, inherent variability in visual interpretation could impact the classification. To minimize this error, we excluded samples with uncertain onset patterns. However, this approach reduced the total number of training samples. Semi-automated annotation methods can be used for future work to enhance reproducibility.

Another limitation is the relatively small number of samples in certain seizure onset categories stratified by brain region. Imbalanced dataset can affect the generalizability, particularly for underrepresented patterns. Expanding the dataset and including more subjects is essential for enhancing the model’s robustness. Additionally, to address overfitting, we employed feature selection technique and subject-wise cross-validation. Techniques such as transfer learning can be investigated in future studies to improve the model generalizability. Addressing these limitations in future research will be crucial for translating these findings into clinically applicable seizure detection and classification tools.

## 5. Conclusion

This study investigated automatic seizure onset pattern classification, with an emphasis on heterogeneity in seizure manifestations in different brain regions and underlying mechanisms. Our rigorous evaluation of the onset classification model demonstrated that machine learning techniques can capture region-specific neural dynamics. Furthermore, this study demonstrated the use of PCN NHP models in seizure onset investigations, contributing to better understanding of region-specific seizure mechanisms. Using advanced computational techniques, this work provides the foundation for understanding the dynamics of seizure networks.

## Figures and Tables

**Table 1.** Number of PCN sessions induced for each animal with the averaged amount of PCN used and the total number of seizures recorded, and onset pattern categorized.

		Number of PCN Sessions	Number of seizures Recorded (Categorized)
**Frontal lobe seizures**	**NHP 1** **NHP 2** **NHP 3**	11 11 16	569 (504) 657 (566) 470 (426)
**Temporal lobe seizures**	**NHP 4** **NHP 5** **NHP 6**	16 9 15	300 (288) 204 (189) 150 (72)

**Table 2.** Classification AUC values calculated with various segment length for different onset pattern classes..

	Onset Segment	Sharp Activity	LAF	Delta Brush	HAS
**AUC (%)**	**2sec Pre- 2sec Post**	84.41	89.82	87.50	94.84
	**2sec Post**	85.45	90.11	89.29	95.49
	**3sec Post**	**90.44**	**92.85**	**92.90**	**97.59**
	**4sec Post**	89.38	92.42	88.34	95.87
	**5sec Post**	88.99	91.89	81.41	94.94

**Table 3.** Performance of the classifier on frontal lobe seizures using subject-wise 3-fold crossvalidation.

	Sharp Activity	LAF	Delta Brush	HAS
**True positive**	370	425	148	250
**False positive**	82	119	57	36
**False negative**	114	75	72	33
**True negative**	921	868	1210	1168
**PPV (%)**	81.86	78.13	72.20	87.41
**Sensitivity (%)**	76.45	85.00	67.27	88.34
**Specificity (%)**	91.82	87.94	95.50	97.01
**Accuracy (%)**	80.23	80.23	80.23	80.23
**F1-Score (%)**	79.06	81.42	69.65	87.87

**Table 4.** Performance of the classifier on independent test set consisting of seizures from the temporal lobe.

	**Sharp Activity**	**LAF**	**Delta Brush**	**HAS**
**True positive**	199	2	51	183
**False positive**	15	0	56	43
**False negative**	48	3	34	29
**True negative**	287	544	408	294
**PPV (%)**	92.99	100	47.66	80.97
**Sensitivity (%)**	80.57	40.00	60.00	86.32
**Specificity (%)**	95.03	100	87.93	87.24
**Accuracy (%)**	79.23	79.23	79.23	79.23
**F1-Score (%)**	86.33	57.14	53.13	83.56

## References

[1] Truccolo W, , Single-neuron dynamics in human focal epilepsy. Nat Neurosci, 2011. 14(5): p. 635–41. 21441925 10.1038/nn.2782 PMC3134302

[2] Lerche H, Drug-resistant epilepsy - time to target mechanisms. Nat Rev Neurol, 2020. 16(11): p. 595–596. 33024326 10.1038/s41582-020-00419-y

[3] Jobst BC, , Intracranial EEG in the 21st Century. Epilepsy Curr, 2020. 20(4): p. 180–188. 32677484 10.1177/1535759720934852 PMC7427159

[4] Velasco AL, , Functional and anatomic correlates of two frequently observed temporal lobe seizure-onset patterns. Neural Plast, 2000. 7(1–2): p. 49–63. 10709214 10.1155/NP.2000.49 PMC2565365

[5] Lee SA, Spencer DD, and Spencer SS, Intracranial EEG seizure-onset patterns in neocortical epilepsy. Epilepsia, 2000. 41(3): p. 297–307. 10714401 10.1111/j.1528-1157.2000.tb00159.x

[6] Spanedda F, Cendes F, and Gotman J, Relations between EEG seizure morphology, interhemispheric spread, and mesial temporal atrophy in bitemporal epilepsy. Epilepsia, 1997. 38(12): p. 1300–14. 9578526 10.1111/j.1528-1157.1997.tb00068.x

[7] Wennberg R, , Preeminence of extrahippocampal structures in the generation of mesial temporal seizures: evidence from human depth electrode recordings. Epilepsia, 2002. 43(7): p. 716–26. 12102674 10.1046/j.1528-1157.2002.31101.x

[8] Perucca P, Dubeau F, and Gotman J, Intracranial electroencephalographic seizure-onset patterns: effect of underlying pathology. Brain, 2014. 137(Pt 1): p. 183–96. 24176980 10.1093/brain/awt299

[9] Lagarde S, , The repertoire of seizure onset patterns in human focal epilepsies: Determinants and prognostic values. Epilepsia, 2019. 60(1): p. 85–95. 30426477 10.1111/epi.14604

[10] Williamson PD, Frontal lobe epilepsy. Some clinical characteristics. Adv Neurol, 1995. 66: p. 127–50; discussion 150-2. 7771297

[11] Bragin DE, , Development of epileptiform excitability in the deep entorhinal cortex after status epilepticus. Eur J Neurosci, 2009. 30(4): p. 611–24. 19674083 10.1111/j.1460-9568.2009.06863.x PMC2776653

[12] Spencer SS, , Morphological patterns of seizures recorded intracranially. Epilepsia, 1992. 33(3): p. 537–45. 1592034 10.1111/j.1528-1157.1992.tb01706.x

[13] Gnatkovsky V, , Fast activity at seizure onset is mediated by inhibitory circuits in the entorhinal cortex in vitro. Ann Neurol, 2008. 64(6): p. 674–86. 19107991 10.1002/ana.21519

[14] Ilyas A, , Ictal high-frequency activity in limbic thalamic nuclei varies with electrographic seizure-onset patterns in temporal lobe epilepsy. Clin Neurophysiol, 2022. 137: p. 183–192. 35216941 10.1016/j.clinph.2022.01.134

[15] Connolly MJ, , Seizure onset and offset pattern determine the entrainment of the cortex and substantia nigra in the nonhuman primate model of focal temporal lobe seizures. Plos one, 2024. 19(8): p. e0307906. 39197026 10.1371/journal.pone.0307906 PMC11356443

[16] Salami P, , Distinct EEG seizure patterns reflect different seizure generation mechanisms. Journal of neurophysiology, 2015. 113(7): p. 2840–2844. 25652916 10.1152/jn.00031.2015 PMC4416632

[17] Prabhu S, , Effect of subthalamic nucleus stimulation on penicillin induced focal motor seizures in primate. Brain Stimul, 2015. 8(2): p. 177–84. 25511796 10.1016/j.brs.2014.10.017

[18] Devergnas A, , The subcortical hidden side of focal motor seizures: evidence from micro-recordings and local field potentials. Brain, 2012. 135(Pt 7): p. 2263–76. 22710196 10.1093/brain/aws134

[19] Sherdil A, , An on demand macaque model of mesial temporal lobe seizures induced by unilateral intra hippocampal injection of penicillin. Epilepsy Res, 2018. 142: p. 20–28. 29547770 10.1016/j.eplepsyres.2018.03.008

[20] Wu D, , Classification of seizure types based on multi-class specific bands common spatial pattern and penalized ensemble model. Biomedical Signal Processing and Control, 2023. 79: p. 104118.

[21] Makaram N, , Automated classification of five seizure onset patterns from intracranial electroencephalogram signals. Clinical Neurophysiology, 2020. 131(6): p. 1210–1218. 32299004 10.1016/j.clinph.2020.02.011

[22] Raghu S, , EEG based multi-class seizure type classification using convolutional neural network and transfer learning. Neural Networks, 2020. 124: p. 202–212. 32018158 10.1016/j.neunet.2020.01.017

[23] Perucca P, Dubeau F, and Gotman J, Intracranial electroencephalographic seizure-onset patterns: effect of underlying pathology. Brain, 2014. 137(1): p. 183–196. 24176980 10.1093/brain/awt299

[24] Garber J, , Guide for the Care and Use of Laboratory Animals (8th ed.). Washington (DC): National Academies Press (US); 2011. , 2011. 21595115

[25] Connolly MJ, , Seizure onset and offset pattern determine the entrainment of the cortex and substantia nigra in the nonhuman primate model of focal temporal lobe seizures. PLoS One, 2024. 19(8): p. e0307906. 39197026 10.1371/journal.pone.0307906 PMC11356443

[26] Connolly MJ, , Effects of acute hippocampal stimulation in the nonhuman primate penicillin model of temporal lobe seizures. Heliyon, 2024. 10(14): p. e34257. 39100434 10.1016/j.heliyon.2024.e34257 PMC11296028

[27] Fisher RS, , ILAE official report: a practical clinical definition of epilepsy. Epilepsia, 2014. 55(4): p. 475–482. 24730690 10.1111/epi.12550

[28] Dolezalova I, , Intracranial EEG seizure onset patterns in unilateral temporal lobe epilepsy and their relationship to other variables. Clin Neurophysiol, 2013. 124(6): p. 1079–88. 23415861 10.1016/j.clinph.2012.12.046

[29] Donos C, , Seizure onset predicts its type. Epilepsia, 2018. 59(3): p. 650–660. 29322500 10.1111/epi.13997

[30] Ogren JA, , Three-dimensional hippocampal atrophy maps distinguish two common temporal lobe seizure-onset patterns. Epilepsia, 2009. 50(6): p. 1361–70. 19054395 10.1111/j.1528-1167.2008.01881.x PMC2773143

[31] Sanabria V, , What we have learned from non-human primates as animal models of epilepsy. Epilepsy & Behavior, 2024. 154: p. 109706. 38518671 10.1016/j.yebeh.2024.109706

[32] Basso MA, , The Future of Nonhuman Primate Neuroscience: Peril or Possibilities? Journal of Neuroscience, 2024. 44(37). 10.1523/JNEUROSCI.1458-24.2024 PMC11391493 39261013

[33] Capitanio JP and Emborg ME, Contributions of non-human primates to neuroscience research. The Lancet, 2008. 371(9618): p. 1126–1135. 10.1016/S0140-6736(08)60489-4 18374844

[34] Sanabria V, , What we have learned from non-human primates as animal models of epilepsy. Epilepsy Behav, 2024. 154: p. 109706. 38518671 10.1016/j.yebeh.2024.109706

[35] Pallud J, , [Animal models to develop surgery of focal epilepsies?]. Neurochirurgie, 2008. 54(3): p. 128–34. 18417167 10.1016/j.neuchi.2008.02.015

[36] Croll L, , Epilepsy in nonhuman primates. Epilepsia, 2019. 60(8): p. 1526–1538. 31206636 10.1111/epi.16089 PMC6779127

[37] Donos C, , Seizure onset predicts its type. Epilepsia, 2018. 59(3): p. 650–660. 29322500 10.1111/epi.13997

[38] Song J-L, Hu W, and Zhang R, Automated detection of epileptic EEGs using a novel fusion feature and extreme learning machine. Neurocomputing, 2016. 175: p. 383–391.

[39] Parvandeh S, , Consensus features nested cross-validation. Bioinformatics, 2020. 36(10): p. 3093–3098. 31985777 10.1093/bioinformatics/btaa046 PMC7776094

[40] Englot DJ and Blumenfeld H, Consciousness and epilepsy: why are complex-partial seizures complex? Progress in brain research, 2009. 177: p. 147–170. 19818900 10.1016/S0079-6123(09)17711-7 PMC2901990

[41] Tinuper P and Bisulli F, From nocturnal frontal lobe epilepsy to sleep-related hypermotor epilepsy: a 35-year diagnostic challenge. Seizure, 2017. 44: p. 87–92. 28027860 10.1016/j.seizure.2016.11.023

[42] Aarabi A and He B, A rule-based seizure prediction method for focal neocortical epilepsy. Clinical Neurophysiology, 2012. 123(6): p. 1111–1122. 22361267 10.1016/j.clinph.2012.01.014 PMC3361618

[43] Bandarabadi M, , Epileptic seizure prediction using relative spectral power features. Clinical Neurophysiology, 2015. 126(2): p. 237–248. 24969376 10.1016/j.clinph.2014.05.022

